# Cumulative Risks from Stressor Exposures and Personal Risk Factors in the Workplace: Examples from a Scoping Review

**DOI:** 10.3390/ijerph18115850

**Published:** 2021-05-29

**Authors:** Mary A. Fox, Richard Todd Niemeier, Naomi Hudson, Miriam R. Siegel, Gary Scott Dotson

**Affiliations:** 1 Department of Health Policy and Management, Risk Sciences and Public Policy Institute, Johns Hopkins Bloomberg School of Public Health, Baltimore, MD 21205, USA; 2 Office of the Director, National Institute for Occupational Safety and Health, Centers for Disease Control and Prevention, Cincinnati, OH 45226, USA; rbn4@cdc.gov; 3 Division of Science Integration, National Institute for Occupational Safety and Health, Centers for Disease Control and Prevention, Cincinnati, OH 45226, USA; iuz8@cdc.gov; 4 Division of Field Studies and Engineering, National Institute for Occupational Safety and Health, Centers for Disease Control and Prevention, Cincinnati, OH 45226, USA; wrm9@cdc.gov; 5 Former CDC/NIOSH, Cincinnati, OH 45226, USA; gary.dotson@cardno.com

**Keywords:** cumulative risk assessment, combined exposure, multiple determinants of health, occupational health, workplace

## Abstract

Protecting worker and public health involves an understanding of multiple determinants, including exposures to biological, chemical, or physical agents or stressors in combination with other determinants including type of employment, health status, and individual behaviors. This has been illustrated during the COVID-19 pandemic by increased exposure and health risks for essential workers and those with pre-existing conditions, and mask-wearing behavior. Health risk assessment practices for environmental and occupational health typically do not incorporate multiple stressors in combination with personal risk factors. While conceptual developments in cumulative risk assessment to inform a more holistic approach to these real-life conditions have progressed, gaps remain, and practical methods and applications are rare. This scoping review characterizes existing evidence of combined stressor exposures and personal factors and risk to foster methods for occupational cumulative risk assessment. The review found examples from many workplaces, such as manufacturing, offices, and health care; exposures to chemical, physical, and psychosocial stressors combined with modifiable and unmodifiable determinants of health; and outcomes including respiratory function and disease, cancers, cardio-metabolic diseases, and hearing loss, as well as increased fertility, menstrual dysfunction and worsened mental health. To protect workers, workplace exposures and modifiable and unmodifiable characteristics should be considered in risk assessment and management. Data on combination exposures can improve assessments and risk estimates and inform protective exposure limits and management strategies.

## 1. Introduction

Most human health risk assessment practices today focus on health effects related to a single stressor. It has been argued, however, that this focus ignores important co-exposures, and therefore, does not adequately assess the exposure-related health risks [1,2]. In addition, it has been recognized that occupationally related diseases are likely multifactorial in nature [3]. Approaches for cumulative risk assessment (CRA) rooted in a multi-stressor framework are designed to tackle these challenges [4,5]. CRA has been part of US Environmental Protection Agency (EPA) pesticide risk assessment work since the passage of the Food Quality Protection Act of 1996. US EPA defined CRA as “combined risks from aggregate exposures to multiple agents or stressors” [6]. This definition reflects the interest in understanding the risks of combined exposures to chemical and non-chemical stressors and their potential interactions [6]. Non-chemical stressors include radiation, viruses, microbes, nutritional status, and economic or psychological stressors, among others [4]. The National Institute for Occupational Safety and Health (NIOSH) has defined mixed exposures as “exposures to either chemical mixtures, different substances at different times, simultaneous exposure to multiple substances, or simultaneous exposure to a chemical substance and another stressor” [7]. This definition is consistent with US EPA’s definition of CRA and is applicable to chemical mixtures, co-exposures to chemicals and other stressors, in addition to exposures to multiple stressors over a defined period [7]. A review of developments in CRA emphasizing human and ecological health identified progress particularly in the problem formulation and exposure assessment steps of the risk assessment process; needs in the area of measures or proxies for non-chemical stressors; and only a few studies on dose-response and risk characterization for CRAs [8].

There is a growing amount of literature and programmatic interest in CRA and worker health. In the 2020 Current Intelligence Bulletin on NIOSH Practices in Occupational Risk Assessment, CRA is characterized as an emerging practice [9]. Williams et al. made the case for advancing CRA beyond consideration of the ambient environment to include personal and occupational risk factors [2]. Schulte et al. described models of work and personal factors combining or interacting in the development of disease [3]. Lentz et al. reviewed aggregate and cumulative risk approaches and presented an aggregate exposure example considering workplace and personal exposures [10]. The Total Worker Health^®^ program incorporates the CRA concept of multiple stressor exposures [11]. While focusing on worker health and safety at the workplace, TWH^®^ recognizes that many issues, including personal risk factors, ambient environmental exposures, and community and social factors, among others, also contribute to worker health and well-being. Fox et al. explored the potential implications and roles for employers and workers in conducting CRA for occupational health [12]. Niemeier et al. considered CRA from the occupational health and safety practitioner’s perspective, noting the need to identify personal risk factors that can modify combined stressor exposure risks and include them in workplace CRA tools [13]. 

This scoping review presents examples from published occupational health research to further help CRA practice by identifying important combined stressor exposures and particularly the non-chemical stressors and personal risk factors to consider for CRA. The results of this review are discussed with attention to the development of potential CRA and management approaches to protect worker health.

## 2. Materials and Methods

**Figure 1:**
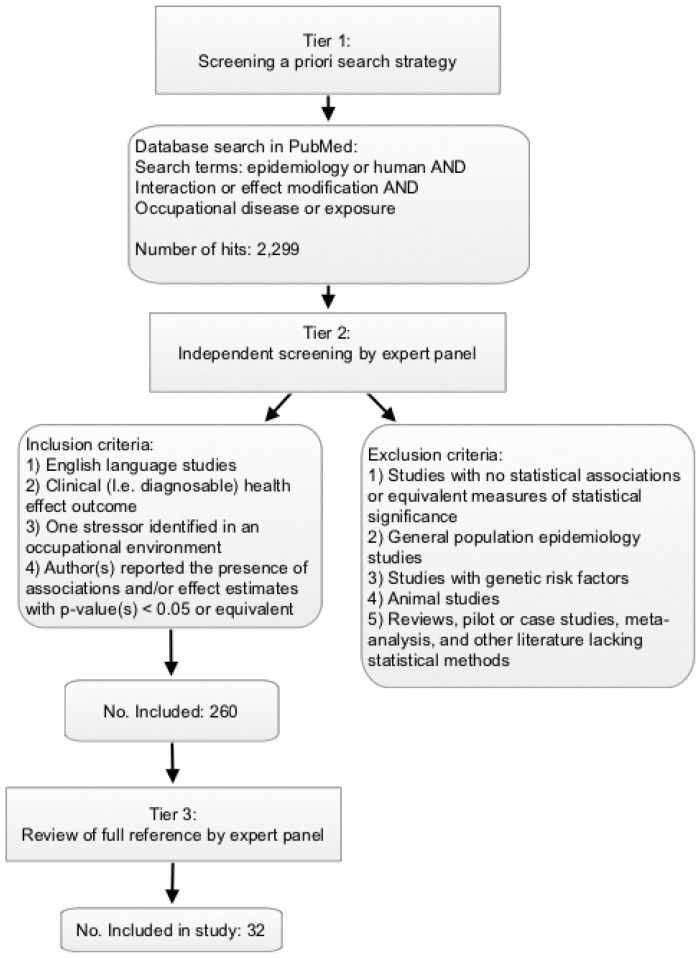
Scoping review schematic.

A three-tiered review approach was developed to identify a sample of relevant scientific literature related to the effects of combined stressors and personal risk factors on a clinical health outcome (Figure 1). Tier 1 screening included an a priori literature search in PubMed, utilizing the assistance of an experienced librarian. The initial search was conducted in July 2014 and updated in December 2014 and September 2017. The following search terms were used: (epidemiology OR human) AND (interaction OR effect modification) AND (occupational disease OR exposure). The results of this literature search were uploaded into Abstrackr (a free online tool for organizing and implementing systematic reviews) for further screening [14,15]. Tier 2 screening included independent screening of abstracts by a panel of multi-disciplinary experts, including two epidemiologists, one toxicologist, and one industrial hygienist. In Tier 2 abstracts were randomly assigned and each abstract was independently screened by two experts using the following inclusion criteria: (1) English language studies only; (2) clinical (i.e., diagnosable) health outcome identified as an outcome measure; (3) at least one stressor (i.e., exposure or risk factor) identified in an occupational environment; (4) author(s) reported the presence of associations and/or effect estimates with *p*-value(s) < 0.05 or similar measures of statistical significance (e.g., non-overlapping 95% confidence intervals). Moreover, the following exclusion criteria were applied to the abstracts: (1) studies that found no statistically significant associations or lacking similar measures of statistical significance; (2) general population (i.e., population-based) epidemiology studies that identified occupational stressors post hoc; (3) studies with genetic risk factors; (4) animal studies; (5) reviews, pilot or case studies, meta-analyses, and other literature lacking statistical methods. Disagreements within the results of Tier 2 screening were resolved by a minimum of three expert panelists involved in the screening effort. Tier 3 screening included a review of the full references that passed Tier 2 screening criteria or those without sufficient information in the abstract to make a decision in Tier 2. The same inclusion/exclusion criteria were applied to Tier 3 screening. Tier 3 screening included a full review of the papers by two experts to further ensure that inclusion/exclusion criteria were met and to identify risk estimates. Disagreements within Tier 3 screening were resolved by the full review team. The most common exclusions at Tier 3 were general population studies and occupational studies lacking risk estimates or clinical health outcomes. Details of the occupational stressor(s), personal risk factor(s), health effect(s), and measure(s) of association or of functional change were extracted for interactions reported in the included articles. It is important to note that the data extracted may not be the main research finding in some cases. 

The extracted data are summarized in narrative form. An analysis ranked by the magnitude of risk helps to identify high priority combined stressor exposures and related health effects as well as elucidate the role of personal risk factors.

## 3. Results

The literature search for Tier 1 screening resulted in the identification of 2299 citations that were uploaded and screened in Abstrackr. Tier 2 review of these citation’s abstracts resulted in disagreements on inclusion status for 414 articles (18%). All disagreements were resolved by additional expert panel review. There were 260 articles (11%) that qualified for Tier 3 review. Tier 3 screening identified the 32 articles (1%) that were included in this study (see Table S1).

The included literature represents various workplaces and occupations, primarily manufacturing, with some extractive and service industries as follows: auto manufacturing (2 articles, 6% of 32), civil servants (3 articles, 9%), cottonseed crushing (1 article, 3%), food production (1 article, 3%), furniture manufacturing (1 article, 3%), herbicide manufacturing (1 article, 3%), lacquer manufacturing (1 article, 3%), metal mining and smelting (5 articles, 16%), military reservists (1 article, 3%), nurses (1 article, 3%), painters (1 article, 3%), pesticide applicators (3 articles, 9%), polyvinyl chloride workers (1 article, 3%), pulp and paper (1 article, 3%), ship building (1 article, 3%), shoe making (1 article, 3%), synthetic leather manufacturing (1 article, 3%), utility workers (1 article, 3%), uranium miners (2 articles, 6%), and various workplaces combined (3 articles, 9%). The included literature is summarized in Table 1.

Most studies evaluated one occupational stressor and one personal risk factor. There was a wide variety of occupational stressor exposures, i.e., chemical, biological, biomechanical strain, physical, and psychosocial. Personal risk factors reported in the included studies were age, food or beverage intake, obesity, pre-existing conditions, sex, and smoking. The literature search captured only a few studies of biological exposures or pre-existing health conditions, or studies of job strain or work stress in combination with other stressor exposures.

The health effects reported in the literature range from changes in function to increased incidence and prevalence of disease affecting many organ systems. Most of the health outcomes were related to respiratory function and disease, including functional decrements in forced expiratory volume in one second (FEV1) and forced vital capacity (FVC) as well as asthma, pulmonary nodules, lung cancer incidence and mortality (11 articles, 34%). Other cancer outcomes were bladder, liver, and prostate (3 articles, 10%). A number of cardio-metabolic outcomes were reported, including hypertension, heart disease, and Type II diabetes (6 articles, 20%). Several papers addressed hearing loss (4 articles, 13%). Musculo-skeletal outcomes included carpal tunnel syndrome and physical functioning disability (2 articles, 6%). Other outcomes included female fertility and menstrual dysfunction, dermatitis, liver enzyme changes, and post-traumatic stress disorder (5 articles, 16%). 

To further characterize the nature and magnitude of combined exposures and risk, all included studies reporting a measure of association (Hazard Ratio, Odds Ratio, Rate Ratio, etc.) were ranked from high to low on the measure reported, see Table 2. Note that some studies have multiple entries in Table 2 if they reported multiple stressor exposures and/or more than one health effect. Six studies did not report a measure of association [18,22,25,36,42,44]. For this analysis, the studies are divided by magnitude of risk roughly into thirds (risk >10, risk of 4 up to 10, and risk of 1.4 up to 4). 

Among occupational stressors associated with the highest risks (>10), solvents and noise are most frequent along with arsenic and metal dust. The most common personal risk factor was smoking (modifiable). The highest risk reported was from a study that examined the interaction between vinyl chloride exposure and history of Hepatitis B infection on liver cancer risk. Health effects in this highest risk category were lung cancer, hearing loss, and high blood pressure.

Stressors associated with risks (4 < 10) were more varied than stressors observed in the highest risk category including radon, arsenic and nickel, dust, certain pesticides, oil, solvents, and increasing wrist velocity. Personal risk factors in this category were age and sex (non-modifiable) and smoking and saturated fat intake (modifiable). Reported health effects were lung cancer, hearing loss, dermatitis, asthma, carpal tunnel syndrome diagnosis and surgery, Parkinson’s disease, and increased female fertility. 

Occupational stressors associated with risk (1.4 < 4) included metals, noise, certain pesticides, solvents, urban air pollutants administering antineoplastic drugs, biomechanical and high physical demand, work stress, psychosocial work strain, and low social support. Personal risk factors in this category were age, family history of prostate cancer, and sex (non-modifiable); and coffee consumption and obesity (modifiable). Health effects reported in this category were cancers (lung, bladder, prostate), coronary heart disease, dermatitis, diabetes mellitus, hearing loss, increased female fertility, menstrual dysfunction, physical function disability, and pulmonary nodules. Three studies assessed stress, psychosocial job strain, and social support at work and one study reported risk from a three-exposure interaction in this category.

## 4. Discussion

### 4.1. Strengths and Limitations

To our knowledge, this is the first review paper that has examined the scientific literature to identify studies with combined stressor (cumulative) exposures where at least one stressor was an occupational exposure. The criteria used to identify these studies were very strict and likely underrepresent the full spectrum of the occupationally based scientific literature that has evaluated combined stressor exposures with personal risk factors. For example, it was decided to exclude population-based epidemiological studies that identified occupational stressors post hoc. However, these criteria were selected to serve as a proof-of-concept approach, providing specific examples that are important to consider in the workplace. Nonetheless, analyses with small sample sizes, which are common among occupational health studies, were likely excluded if low power inhibited statistically significant results. Additionally, the exclusion of studies that did not identify clinical health outcomes meant that studies exploring many psychosocial or genetic outcomes or continuous measures of health function were set aside. Similarly, studies evaluating genetic risk factors in combination with occupational stressors were excluded from the present analysis but reserved for separate evaluation. The need for additional reviewers with genetic expertise motivated this decision. Evaluating these bodies of work could also provide insights into potential CRA and management approaches to protect worker health. 

In any body of literature, there will be variability in the conduct (exposure and outcome measurement, analytical methods) of each study as well as the reporting of results. For example, only a subset of the papers evaluated here included further description of the types of interactions observed, i.e., additive, super-additive, or less than multiplicative [26,39,40,43,47]. Variability within the literature will pose a challenge to efforts to translate the quantitative data for application in a risk assessment context. Previous work discussed the potential ways to develop CRA for occupational safety and health (OSH), including research funding, expert panel workshops, trainings, and agency guidance [12,13]. To advance CRA for OSH, it will be critically important to have guidance on the identification, evaluation, and analysis of data relevant to cumulative risk topics such as those identified above. US EPA’s guidance on age-dependent adjustments for early-life exposure to mutagenic carcinogens and the related data analysis provides a useful example [48,49].

Despite the limitations of the strict inclusion criteria, the literature reviewed here indicates that there are important topics that deserve further attention in the occupational CRA context, such as:

- Controlling noise and solvent exposures;
- Understanding the dose–response relationship and interactions of combined stressors;
- Considering the influence of common unmodifiable characteristics, including age, sex, and pre-existing health conditions;
- Understanding stressor exposures that are likely widespread, such as job strain, or those that may increase in likelihood or intensity in coming years, such as heat (particularly for outdoor workers);
- Understanding combination stressor exposures across more occupational categories (the present review captured predominantly manufacturing);
- Identifying personal characteristics, behaviors, community or psychosocial factors that mitigate or reduce risk.

### 4.2. Applying Combined Exposure Data to Improve Worker Health 

This literature review provides examples that combined stressor exposures, both chemical and non-chemical, along with personal risk factors, influence worker health and shows the continuing importance of existing workplace well-being efforts. About one-third of the literature reviewed evaluated effects of smoking and an occupational stressor exposure, highlighting the critical role of smoking cessation programs to improve worker health. Several papers evaluated age as an important non-modifiable personal factor in exposure and risk, a topic of current interest for the Total Worker Health^®^ program in its National Center for Productive Aging and Work [50]. Hearing loss being a common outcome among the studies finding very high risk is consistent with OSHA and NIOSH concerns highlighted in a recent safety and health information bulletin [51].

Looking beyond current programs, this literature offers indications on how considering cumulative exposure and risk data could inform occupational safety and health risk assessments and risk management approaches. The literature reviewed illustrates that there are many different combined stressor and personal risk factor exposures, affecting various workplaces and types of workers. Based on the findings of this study, combined stressor and personal risk factor exposures increase the risk of disease and functional deficits in most cases in the published literature and include modifiable and unmodifiable behaviors and characteristics. To protect the health of workers, both modifiable and unmodifiable behaviors and characteristics should be considered in risk assessment and risk management. Assessment and management approaches will depend on the work and workplace context and type of exposure. Since the assessment and management of combined exposures are likely resource intensive, careful consideration of the boundaries and scale of these efforts are important. These considerations in both the occupational and non-occupational environment have been previously discussed [13,52,53]. In addition, as with any risk assessment, defining the scope and focus via an in-depth problem formulation is recommended prior to beginning the study to ensure that the findings can be applied in guiding decision-making in selecting appropriate risk management steps [1].

The literature reviewed suggests several ways combined stressor and personal risk factor exposure and risk information can be applied:

1. When establishing exposure limits through the risk assessment and management process:
  - Different limits can be set in workplaces with the demonstrated potential for co-exposures to other stressors. For example, methylene chloride is metabolized in the human body to carbon monoxide (CO). NIOSH published a Recommended Exposure Limit (REL) for methylene chloride that was adjustable based on the presence and concentration of CO, because both exposures contribute to the formation of carboxyhemoglobin, which can impair delivery of oxygen to the tissues of the body and lead to abnormalities in functions of the central nervous system [54].
  - Workplace exposure limits can be adjusted in consideration of exposures to the same chemical outside the workplace, e.g., aggregate exposure and relative source contribution, as described in Lentz et al. [10].
2. When estimating risks:
  - Risk estimates could account for common unmodifiable characteristics, such as age, sex, and race/ethnicity. Both disease occurrence and disease risk factors can vary by these unmodifiable characteristics due to biological mechanisms and social determinants of health. Age and sex were identified as important determinants of risk in several studies [21,24,25,29,42,44,45]; the interpretation and contribution of these factors varied in the context of each study and no overarching conclusions can be reached. One example of an existing risk methodology that takes age into account is EPA’s age-dependent adjustment factors in estimating child cancer risk from early-life exposures to mutagens [48,49].
  - Established risk assessment practices typically apply an additivity assumption when combining exposures. Although the examples found in this review are few, some studies reported greater than additive interactions, suggesting that defaulting to additivity may underestimate risk in some situations.
3. When selecting risk management options:
  - An understanding of cumulative exposures can inform decisions among the hierarchy of controls if many workers have an unmodifiable characteristic or pre-existing condition that contributes to increased risk when co-exposure occurs.

Both the American Council of Government Industrial Hygienists (ACGIH) and OSHA/NIOSH have published guidance and information on hearing loss from exposures to noise and certain chemicals and drugs, including the need to consider such exposures in risk management choices (administrative or engineering controls, etc.) and provision of routine health monitoring [51,55].

### 4.3. COVID-19 and Worker Risk

Early indications from ongoing research on COVID-19 reveals the potential for combined exposure and risk. In the general population, increased COVID-19 disease risk has been found across personal risk factors, including age and pre-existing disease [56,57,58]. Essential workers in healthcare, social work, other personal care jobs, and food production are among those essential work categories at higher risk [59,60]. We anticipate combined stressor exposure and CRA to be a frequent theme of emerging research on essential workers. Such research would address gaps observed above, e.g., we found only one article related to infectious disease (Hepatitis B) and one article each representing healthcare and food production workers. 

## 5. Conclusions

CRA frameworks are designed to allow a more holistic evaluation of exposures and risk factors across the multiple domains of everyday life: work; home/personal; ambient environment; and community [12]. Ongoing work at NIOSH including exposome research (i.e., the measure of all the exposures of an individual in a lifetime and how those exposures relate to health) and TWH^®^ are potential collaborative opportunities to further develop CRA and management methods and practices for worker health protection [61,62]. However, a coordinated research, policy, and industrial hygiene training and practice effort will be needed to develop and deploy CRA and management tools for worker health.

## Figures and Tables

**Table 1:** Summary of included literature.

Stressor or Risk Factor Combination	Number of Articles	Work-Related Stressor Exposure(s) ^a^	Personal Risk Factors ^a^	Health Effect(s) ^a^	References
Chemical and personal	16	Solvents, solvents and oil, soluble nickel, metal ore dust, urban air, arsenic and metals from smelting (2), pesticides (3), mixed dust (2), wood dust, CO, TCDD, antineoplastic drugs	Age (4), family history, sex, saturated fat intake, smoking or not smoking (9)	Asthma, bladder cancer, dermatitis, lung cancer, lung function, pulmonary nodules, diabetes, Parkinson’s, heart disease, liver damage, female fertility, menstrual dysfunction	[16,17,18,19,20,21,22,23,24,25,26,27,28,29,30,31]
Chemical and physical	4	Solvents and noise (4)		Hypertension (2), hearing loss (2)	[32,33,34,35]
Chemical and biological	2	Cotton dust, vinyl chloride	Atopy, HepB surface antigen positive	Lung function, liver cancer	[36,37]
Physical and personal	2	Noise (2)	Smoking (2)	Hearing loss	[38,39]
Physical exertion and personal	1	Increasing wrist velocity	Sex	Carpal tunnel syndrome diagnosis or surgery	[40]
Radon and personal	3	Radon (3)	Age (3)	Lung cancer	[41,42,43]
Psycho-social and personal	2	Military authority, work stress	Sex, sex and obesity	PTSD, diabetes	[44,45]
Physical exertion and psycho-social	2	High physical demand and job strain, biomechanical exposure, and low social support		Heart disease, physical functioning disability	[46,47]

^a^ Number of articles indicated in parentheses if there is more than one for the exposure or outcome listed. Abbreviations: CO: carbon monoxide; CVD: cardiovascular disease; HepB: hepatitis B; PTSD: post-traumatic stress disorder; TCDD: 2,3,7,8 tetrachlorodibenzo-p-dioxin.

**Table 2:** Studies ranked by magnitude of risk (high to low).

Measure of Association (CI)	Occupational Stressor 1	Occupational Stressor 2	Personal Risk Factor	Health Effect	Reference
OR = 184.5 (15–infinity)	Vinyl chloride		HBsAg +	Liver cancer	[37]
RR = 47 (5.3–415.7)	Cumulative dust		Smoking	Lung cancer	[30]
OR = 29.6 (2.6–335.6)	As >100 mg/m^3^-yrs		Smoking	Lung cancer ^	[26]
OR = 21.5 (5–26)	Mixed solvents (styrene, toluene)	Noise		Hearing loss	[35]
OR = 20.2 (3.8–26)	Mixed solvents (hexane, toluene)	Noise		Hearing loss	[35]
OR = 14.22 (3.21–40.84)	Organic solvents	Noise		High blood pressure	[32]
OR = 13.5 (1.5–117.8)	Organic solvents	Noise		High blood pressure	[33]
RR = 10.9 (4.1–28.9)	Toluene	Noise		Hearing loss	[34]
OR = 10.4 (1.2–86.6)	As 15 to <100 mg/m^3^-yrs		Smoking	Lung cancer ^	[26]
SRR = 9.5 (3.7–25)	Radon		Smoking	Lung cancer ^	[43]
OR = 7.9 (1–63.1)	As 0.25 to <15 mg/m^3^-yrs		Smoking	Lung cancer ^	[26]
OR = 7.8 (4.7–13)	Noise		Smoking	Hearing loss	[38]
OR = 7.17 (2.64–19.5)	Oil	Solvent		Dermatitis	[31]
OR = 6.45 (1.07–38.98)	Dust exposure		Female sex	Asthma	[24]
Adjusted IRR = 6.37 (3.64–11.3)	Increasing wrist velocity		Sex	Carpal tunnel syndrome surgery +	[40]
RR = 5.81 (2.44–10.68)	Rn >5 WLM		Age of first exposure 30–39	Lung cancer	[42]
OR = 5.8 (2.3–14.6)	Rotenone		Higher saturated fat intake	Parkinson’s disease	[19]
OR = 5.25 (1.66–16.6)	Solvent		Smoking	Increased female fertility	[27]
RR = 5.1 (1.3–20.5)	Soluble Nickel		Smoking	Lung cancer	[16]
RR = 4.43 (2.1–7.65)	Rn >5 WLM		Age of first exposure >40	Lung cancer	[42]
OR = 4.2 (1.5–12)	Paraquat		Low saturated fat intake	Parkinson’s disease	[19]
Adjusted IRR = 4.11(2.61–6.48)	Increasing wrist velocity		Sex	Carpal tunnel syndrome diagnosis +	[40]
HR = 3.63 (1.08–12.22)	High physical work demand	Low social support		Coronary heart disease	[46]
PR = 3.6 (2.4–5.4)	Metal exposure		Smoking	Diabetes mellitus	[28]
OR = 3.43 (1.61–7.32)	Administering antineoplastic drugs		Age	Menstrual dysfunction	[29]
OR = 3.28 (1.10–9.92)	Oil	Solvent	Age > 35	Dermatitis	[31]
RR = 3.03 (1.46–6.29)	Pesticide		Smoking	Bladder cancer	[20]
RR = 2.97 (*p* < 0.001)	Rn WLM		Age < 60	Lung Cancer	[41]
OR = 2.66 (1.42–4.99)	Solvent		Coffee	Increased female fertility	[27]
RR = 2.14 (1.02–4.52)	Urban air pollutants		Smoking	Pulmonary nodules	[23]
HR = 2.01 (1.06–3.92	Work Stress		Female sex, Obesity	Type 2 diabetes	[45]
RR = 1.91 (1.23–2.95)	Coumaphos		Family history of prostate cancer	Prostate cancer	[17]
RR = 1.91 (1.61–2.26)	Biomechanical	Psychosocial job strain		Physical function disability ^	[47]
OR = 1.94 (1.31–2.88)	Noise		Smoking	Hearing loss +	[39]
PR = 1.4 (1.0–2.3)	Metals		Smoking	Diabetes mellitus	[28]

+ Indicates an interaction characterized as additive. ^ Indicates an interaction characterized as greater than additive and less than multiplicative. Abbreviations: As: arsenic; CI: confidence interval; dB: decibel; Expo: exposure; FEV1: forced expiratory volume 1 s; FVC: forced vital capacity; HR: hazard ratio; HD: heart disease; HepB: Hepatitis B; HBsAg: Hepatitis B surface antibody; Hz: Hertz; IRR: incidence rate ratio; mg/m^3^: milligram per cubic meter; OR: odds ratio; PR: prevalence ratio; Rn: radon; RR: relative risk; SRR: standardized rate ratio; WLM: working level months; yrs: year.

## Supplementary Materials

The following are available online at https://www.mdpi.com/article/10.3390/ijerph18115850/s1, Table S1: Summary of included studies.
