# Supplementary material for: Cumulative Risks from Stressor Exposures and Personal Risk Factors in the Workplace: Examples from a Scoping Review
Source: Int J Environ Res Public Health. 2021 May 29;18(11):5850. doi: 10.3390/ijerph18115850 (PMC8199163; doi:10.3390/ijerph18115850)
Supplement: Supplementary file 1 [file ijerph-18-05850-s001.zip › ijerph-1206464-SI.pdf]

Table S1. Summary of included studies

Note: Studies may be presented in more than one row if multiple stressor metrics or interaction health effects were reported.

**Reference**	**Stressor 1**	**Stressor 2**	**Personal Risk Factor**	**Interaction health effect**	**Measure of Association Risk Estimate (CI or p-value)**
**Chemical and Personal**					
Andersen et al. [1996] PMID: 8943837	Soluble Nickel		Smoking	Lung cancer	RR=5.1 (1.3-20.5) worker smokers vs worker non-smokers
Christensen et al. [2010] PMID:20056581	Coumaphos		Family history of prostate cancer	Prostate cancer	RR = 1.91 (1.23-2.95)
Coenraads et al. [1985] PMID:3158471	Oil exposure	Solvent exposure	Age>35 (Interpreted as increased duration of exposure)	Dermatitis	OR = 7.17 (2.64-19.5) Oil*Sol<br>OR = 3.28 (1.10-9.92) Oil*Sol*Age>35
Fu et al. [1992] PMID:1449657	Cumulative metal ore dust exposure		Smoking	Lung cancer mortality	RR = 47 (5.3-415.7)^
Henneberger et al. [1993] PMID:8317817	Pulp mill work	Irritant gasses (chlorine, sulfur dioxide)	Smoking	Decreased FEV1, FVC and FEV1/FVC with two exposures, increased FEV1, FVC and FEV1/FVC with 3-exposure interaction	FEV1 (2-exposures)<br>Ranged from -7.2 to -329.8 ml (p<0.05)<br>FVC (2-exposures)<br>-6.2 ml (p<0.01)<br>FEV1/FVC (2-exposures)<br>-0.39 ml (p<0.05)<br>FEV1 (3-exposures)<br>20.9 ml (p<0.01)<br>FVC (3-exposures)<br>15.9 ml (p<0.05)<br>FEV1/FVC (3-exposures)<br>0.28 ml (p<0.01)
Jarup and Pershagen [1991] PMID:1951260	Arsenic exposure – smelter workers >100 mg/m^3 yrs		Smoking	Lung cancer	OR = 29.6 (2.6-335.6)
Jarup and Pershagen [1991] PMID:1951260	Arsenic exposure – smelter workers 15 to <100 mg/m^3 yrs		Smoking	Lung cancer	OR = 10.4 (1.2-86.6)
Jarup and Pershagen [1991] PMID:1951260	Arsenic exposure – smelter workers 0.25 to <15 mg/m^3 yrs		Smoking	Lung cancer	OR = 7.9 (1-63.1)

Kamel et al. [2014] PMID:24120951	Rotenone or paraquat Pesticide exposure		Saturated fat intake	Parkinson's disease	OR = 5.8 (2.3-14.6) Rotenone*Higher saturated fat intake<br>OR = 4.2 (1.5 – 12) Paraquat*Lower PUFA intake
Koutros et al. [2016] PMID:26411407	Multiple pesticide exposures. Not a mixture study, pesticide exposures were examined one at a time		Stratified by smoking status	Bladder cancer incidence	RR = 3.03 (1.46 – 6.29)<br>Interaction of high use of imazethapyr and never smoking
Neuberger et al. [1999] PMID:10585016	TCDD		Age	Chronic liver damage (altered liver enzymes)	BE = -3.165 (SGOT) (p<0.034)<br>BE = -3.001 (SGPT) (p<0.047)
Osterman et al. [1989] PMID:2818959	Respirable dust exposure		Smoking	Reduced pulmonary function	FEV1 -8.2 ml/year of employment p<0.03<br>FVC -9.4 ml/year of employment p<0.01
Sallmen et al. [2008] PMID:17989205	Solvent exposure		Smoking or Coffee	Increased female fertility (Fecundability density ratio)	OR=5.25 (1.66-16.6) Sol*Smoking<br>OR=2.66 (1.42-4.99) Sol*Coffee<br>Authors note small numbers underlying these interactions
Sancini et al. [2010] PMID:20430373	Urban air pollutants		Smoking	Pulmonary nodules	RR = 2.14 (1.02-4.52)<br>Note: smoker traffic officer vs. smoker control<br>RR = 2.09 (1.19-3.66)<br>Note: smoker traffic officer vs. non-smoker traffic officer
Schlunssen et a. [2002] PMID:11802471	Dust exposure		Female sex	Physician diagnosed asthma	OR = 6.45 (1.07-38.98)<br>High dust exposure and female sex
Shortridge et al. [1995] PMID:8564939	Administration of antineoplastic drugs		Age	menstrual dysfunction	OR = 3.43 (1.61-7.32)
Stern et a. [1988] PMID:2461656	Carbon monoxide		Age	Arteriosclerotic heart disease, diagnosed at death in male tunnel and bridge workers	5.5% per year of age increase in risk of arteriosclerotic HD comparing tunnel to bridge workers from age 45 on (p<0.015)
Yang et al. [2017] PMID:27418044	Metal exposure- Mining production		Smoking	Diabetes mellitus	PR = 1.4 (1.0 – 2.3)
Yang et al. [2017] PMID:27418044	Metal exposure- smelting/refining		Smoking	Diabetes mellitus	PR = 3.6 (2.4 – 5.4)

**Chemical and Physical**					
Attarchi et al. [2013] PMID:22715117	Mixed organic solvent exposure	Noise		Prevalence of high blood pressure	OR = 14.22 (3.21-40.84)
Chang et al. [2009] PMID:19502770	Organic solvents	Noise		Hypertension	OR = 13.5 (1.5-117.8)
Morata et al [1993] PMID:8235513	Toluene	Noise		Hearing loss	RR=10.9 (4.1-28.9)
Sliwinska-Kowalska [2005] PMID:21783525	Solvents	Noise		Hearing loss	OR=20.2 (3.8 – 26) Noise, hexane and toluene<br>OR=21.5 (5 – 26) Noise, styrene and toluene
**Chemical and Biological**					
Jones et al. [1980] PMID:7426464	Cotton (linter) dust		Atopy	FEV1 decline	>150 ml (p=0.05)
Wong et al. [2003] PMID:12708141	Vinyl chloride		HBsAg Positive HepB surface antigen	Liver cancer	OR=184.5 (15-infinity)
**Physical and Personal**					
Hornung et al. [1998] PMID:9415577	Working level months in uranium mining- exposure to radon decay products		Age (<60)	Lung Cancer	RR=2.97 (p<0.001)
Howe et al. [1986] PMID:3461198	Radon exposure >5 working level months		Age of first exposure 30-39	Lung cancer mortality	RR=5.81 (2.44-10.68)
Howe et al. [1986] PMID:3461198	Radon exposure >5 working level months		Age of first exposure >40	Lung cancer mortality	RR=4.43 (2.1-7.65)
Pouryaghoub et al. [2007] PMID:17605828	Noise		Smoking	Hearing loss	OR= 7.8 (4.7-13) (hearing threshold diff of 30dB b/t 4000-10000Hz)<br>OR= 7.7 (4.9-12.1) (25 dB at 4000 Hz)
Schubauer-Berigan et al. [2009] PMID:19208723	Radon		Smoking	Lung cancer mortality	SRR= 9.5 (3.7-25)<br>Lung cancer mortality rate ratio miners at highest WLM and >1pack/day
Tao et al. [2013] PMID:23412581	Noise exposure		Smoking	High frequency hearing loss	OR= 1.94 (1.31-2.88)
**Physical Exertion and Psychosocial or Personal**					

Clays et al. [2016] PMID:27577590	High physical work demand	Low social support at work		Coronary heart disease incidence	HR=3.63 (1.08-12.22)
Sabbeth et al. [2013] PMID:24796708	Biomechanical exposure	Psychosocial job strain (high demand, low control)		Physical functioning disability post-retirement	RR=1.91 (1.61-2.26)
Heilskov-Hansen et al. [2017] PMID:27030204	Increasing wrist velocity		Sex	Carpal tunnel syndrome diagnosis	Crude IRR= 2.84 (1.94-4.16)<br>Adjusted IRR=4.11(2.61-6.48)<br>Additive interaction: female sex, wrist velocity and mean power frequency
Heilskov-Hansen et al. [2017] PMID: 27030204	Increasing wrist velocity		Sex	Carpal tunnel syndrome surgery	Crude IRR= 3.31 (2.07-5.3)<br>Adjusted IRR= 6.37 (3.64-11.3)<br>see note above
**Psychosocial and Personal**					
Cohen et al. [2016] PMID:26899583	Military authority		Sex	PTSD	BE=-2.37 (p<0.01)
Heraclides et al. [2012] PMID: 21593804	Work Stress		Female sex, Obesity	Type 2 diabetes	HR =2.01 (1.06-3.92

^Confidence interval calculated from data in article.

Abbreviations: BE: beta estimate; CI: confidence interval; dB: decibel; FEV1: forced expiratory volume 1 second; FVC: forced vital capacity; HR: hazard ratio; HD: heart disease; HepB: Hepatitis B; HBsAg: Hepatitis B surface antibody; Hz: Hertz; IRR: incidence rate ratio; OR: odds ratio; PR: prevalence ratio; PTSD: post-traumatic stress disorder; PUFA: Poly-unsaturated fatty acids; RR: relative risk; SGOT: serum glutamic-oxaloacetic transaminase; SGPT: serum glutamic-pyruvic transaminase; Sol: solvent; SRR: standardized rate ratio; TCDD: 2,3,7,8 tetrachlorodibenzo-p-dioxin; VC: vinyl chloride; WLM: working level months; yrs: years
